# Analysis of Psychiatric Disorders by Age Among Children Following a Mass Terrorist Attack in Nice, France, on Bastille Day, 2016

**DOI:** 10.1001/jamanetworkopen.2022.55472

**Published:** 2023-02-03

**Authors:** Florence Askenazy, Nicolas Bodeau, Ophélie Nachon, Mélanie Gittard, Michèle Battista, Arnaud Fernandez, Morgane Gindt

**Affiliations:** 1 Service Universitaire de psychiatrie de l’enfant et de l’adolescent, Hôpitaux pédiatriques de Nice, Centre Hospitalier Universitaire-Lenval, Nice, France; 2 CoBTeK (Cognition-Behaviour-Technology) Lab, Université Cote d’Azur, Nice, France; 3 Paris School of Economics, Centre International de Recherche sur l’Environnement et le Developpement and Ecole Nationale des Ponts et Chaussées, Paris, France

## Abstract

This cross-sectional study examines psychiatric disorders among children after a mass terrorist attack in Nice, France, in 2016.

## Introduction

Terrorist attacks have increased in recent decades.^1^ It has been estimated that more than 32 million children have been affected by complex humanitarian emergencies (eg, civil wars, terrorism, and the Ukraine conflict).^1^

Between 2015 and 2016, France was affected by mass terror attacks. There were approximately 30 000 people—tourists, locals, and many families—in Nice, France, during the 2016 terrorist attack. In this attack, 86 people died, including 15 children.^2^ The frequency of pediatric posttraumatic stress disorder (PTSD) after a terrorist attack ranges from 50.6% to 75.2%.^3,4^ A pediatric consultation center was created at the Children’s University Hospital in Nice, France. Three years later, more than 4700 consultations have been performed.

We report preliminary results of a prospective longitudinal epidemiologic cohort. This study of consecutive outpatients aims to assess the frequency of PTSD and other *Diagnostic and Statistical Manual of Mental Disorders* (Fifth Edition) diagnoses in children. We hypothesize that there is no association between age and PTSD frequency.

## Methods

This cross-sectional study was approved by the National Ethics Committee and followed the Strengthening the Reporting of Observational Studies in Epidemiology (STROBE) reporting guideline. Parents provided written informed consent, and the children provided verbal agreement.

Participants younger than 18 years during the attack were consecutively recruited from November 21, 2017, to November 22, 2019 (eFigure in Supplement 1). The setting is a consultation center solely dedicated to the children exposed to the attack. They were divided into 3 age groups: 0 to 6 years, 7 to 12 years, and 13 to 18 years.

Diagnostic Infant Preschool Assessment (DIPA; 0 to 6 years) and Kiddie Schedule for Affective Disorders and Schizophrenia (K-SADS; 7 to 18 years) were used. DIPA and K-SADS were semistructured interviews that lasted 1 hour and assessed DSM-5 diagnoses.

Descriptive statistics were used for continuous variables, and Fisher exact tests were used to compare the frequency of psychiatric diagnoses across age. Statistical analysis was performed from January to June 2022 using R version 4.2.2 (R Project for Statistical Computing). All tests were 2-tailed, and a *P* value of <.05 was considered significant. In a first step, a descriptive analysis was performed, and frequencies were computed for qualitative variables, and mean, SD for quantitative variables. In a second step, a bivariate analysis was used based on a priori hypotheses. Fisher exact tests were used to compare the frequency of psychiatric diagnoses across age brackets.

## Results

This study included 271 outpatients among the 1217 (22%) registered after the attack, with a mean (SD) age of 9.0 (4.1) years (range, 1-17 years) and 139 males (51%). The mean (SD) number of diagnoses per child was 2.4 (1.8). The frequencies of the *Diagnostic and Statistical Manual of Mental Disorders* (Fifth Edition) diagnoses per age group are presented in the Figure. Overall, 167 of 270 participants (62%) had PTSD, 212 of 270 (79%) had anxiety disorders, 23 of 270 (9%) had major depressive disorder (MDD), 48 of 267 (18%) had impulse control disorders (ICD), and 89 of 268 (33%) had attention deficit disorder (ADD) or attention deficit hyperactivity disorder (ADHD).

**Figure.**
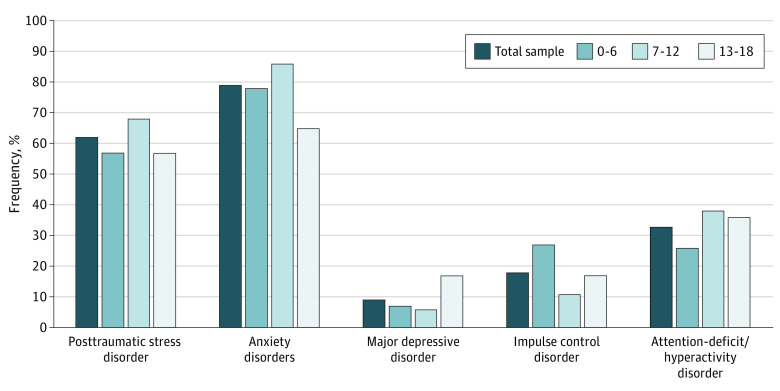
Psychiatric Disorder Frequencies Among Children and Adolescents per Age Categories

Anxiety disorders included specific phobia (151 of 270 [56%]), separation anxiety disorder (77 of 270 [29%]), generalized anxiety disorder (56 of 270 [21%]), social anxiety disorder (15 of 270 [6%]), obsessive-compulsive disorder (3 of 270 [1%]). ICD included intermittent explosive disorder (25 of 270 [9%]), oppositional defiant disorder (44 of 270 [16%]), conduct disorder (5 of 268 [2%]), ADD (43/270 [16%]), and ADHD (30 of 270 [11%]). Some participants fulfilled the criteria for several disorders; thus the sum of diagnoses was greater than the total number of patients.

The association between DSM-5 diagnoses and age groups are presented in the Table. PTSD frequency was not associated with the age groups. A significant association of age was observed for the diagnoses of anxiety disorders.

**Table.** Association of Age Groups With Study Outcomes^a^

Characteristics	Participants, No. (%)	No. (%) [residual]			*P* value
		[0-6]	[7-12]	[13-18]	
Sample size					
All	271	91	120	60	
Female	132 (49)	43 (47)	55 (46)	34 (57)	
Male	139 (51)	48 (53)	65 (54)	26 (43)	
General statistics, mean (SD)					
Age, y	9.0 (4.1)	4.2 (1.6)	9.9 (1.6)	14.3 (1.5)	
No. of diagnosis	2.4 (1.8)	2.1 (2.2)	2 (1.6)	1.9 (1.65)	
PTSD	167 (62)	52 (57)	80 (68)	34 (57)	.16
Anxiety disorders	212 (79)	71 (78)	101 (86)	40 (66)	.005
Specific phobia	151 (56)	63 (69) [3.13]	63 (54) [−0.62]	24 (40) [−2.83]	.002
Separation anxiety	77 (29)	30 (33)	37 (32)	10 (17)	.054
Generalized anxiety	56 (21)	4 (4) [−4.76]	35 (30) [3.20]	17 (28) [1.61]	<.001
Social anxiety disorder	15 (6)	8 (9)	6 (5)	1 (2)	.18
OCD	3 (1)	2 (2)	0	1 (2)	.24
Major depressive disorder	23 (9)	6 (7)	7 (6)	10 (17)	.06
Impulse control disorders	48 (18)	25 (27)	13 (11)	10 (17)	.01
Intermittent explosive disorder	25 (9)	14 (15) [2.44]	5 (4) [−2.50]	6 (10) [0.20]	.02
Oppositional defiant disorder	44 (16)	25 (27) [3.50]	13 (11) [−2.06]	6 (10) [−1.52]	.003
Conduct disorder	5 (2)	0 [−1.63]	0 [−1.98]	5 (8) [4.22]	<.001
ADH/D	89 (33)	23 (26)	44 (38)	22 (36)	.14
Attention deficit disorder	43 (16)	16 (18)	20 (17)	6 (10)	.40
Attention deficit with hyperactivity disorder	30 (11)	14 (15)	14 (12)	2 (3)	.05

Abbreviations: ADH/D, attention deficit with and without hyperactivity; OCD, obsessive compulsive disorder; PTSD, posttraumatic stress disorder.

^a^ This table gives the results from the Fisher Tests 5%. Results are given with the residuals when the difference was significant (meaning *P* value < .05).

## Discussion

In this cross-sectional study, the high frequency of PTSD was not associated with age, while most of the other disorders were. Studies using DSM-5 showed a high rate of PTSD in youths after a terrorist attack.^3,4^ Studies using *Diagnostic and Statistical Manual of Mental Disorders* (Fourth Edition, Text Revision) did not consider developmental specificities and found a lower rate of PTSD.^5^ Our results are consistent with the literature underlying the link between age and anxiety disorders in this population.^6^ The strength is the homogeneity of the population. This study had limitations. For example, we were unable to observe anxiety and PTSD frequencies within the cohort prior to July 14, 2016. These results evidence the need to create pediatric referral centers without age limitations and develop public health policies to care for pediatric PTSD after mass terrorist attacks.

## Notes

Supplement 1. **eFigure.** Flow Diagram

Supplement 2.
Data Sharing Statement
