# Supplementary material for: Analysis of Psychiatric Disorders by Age Among Children Following a Mass Terrorist Attack in Nice, France, on Bastille Day, 2016
Source: JAMA Netw Open. 2023 Feb 3;6(2):e2255472. doi: 10.1001/jamanetworkopen.2022.55472 (PMC9898818; doi:10.1001/jamanetworkopen.2022.55472)
Supplement: Supplement 1. — eFigure. Flow Diagram [file jamanetwopen-e2255472-s001.pdf]

Supplemental Online Content

Askenazy F, Bodeau N, Nachon O, et al. Analysis of psychiatric disorders by age among children following a mass terrorist attack in Nice, France, on Bastille Day, 2016. *JAMA Netw Open*. 2023;6(2):e2255472. doi:10.1001/jamanetworkopen.2022.55472

eFigure. Flow diagram

This supplemental material has been provided by the authors to give readers additional information about their work.

© 2023 Askenazy F et al. *JAMA Network Open.*

eFigure. Flow Diagram

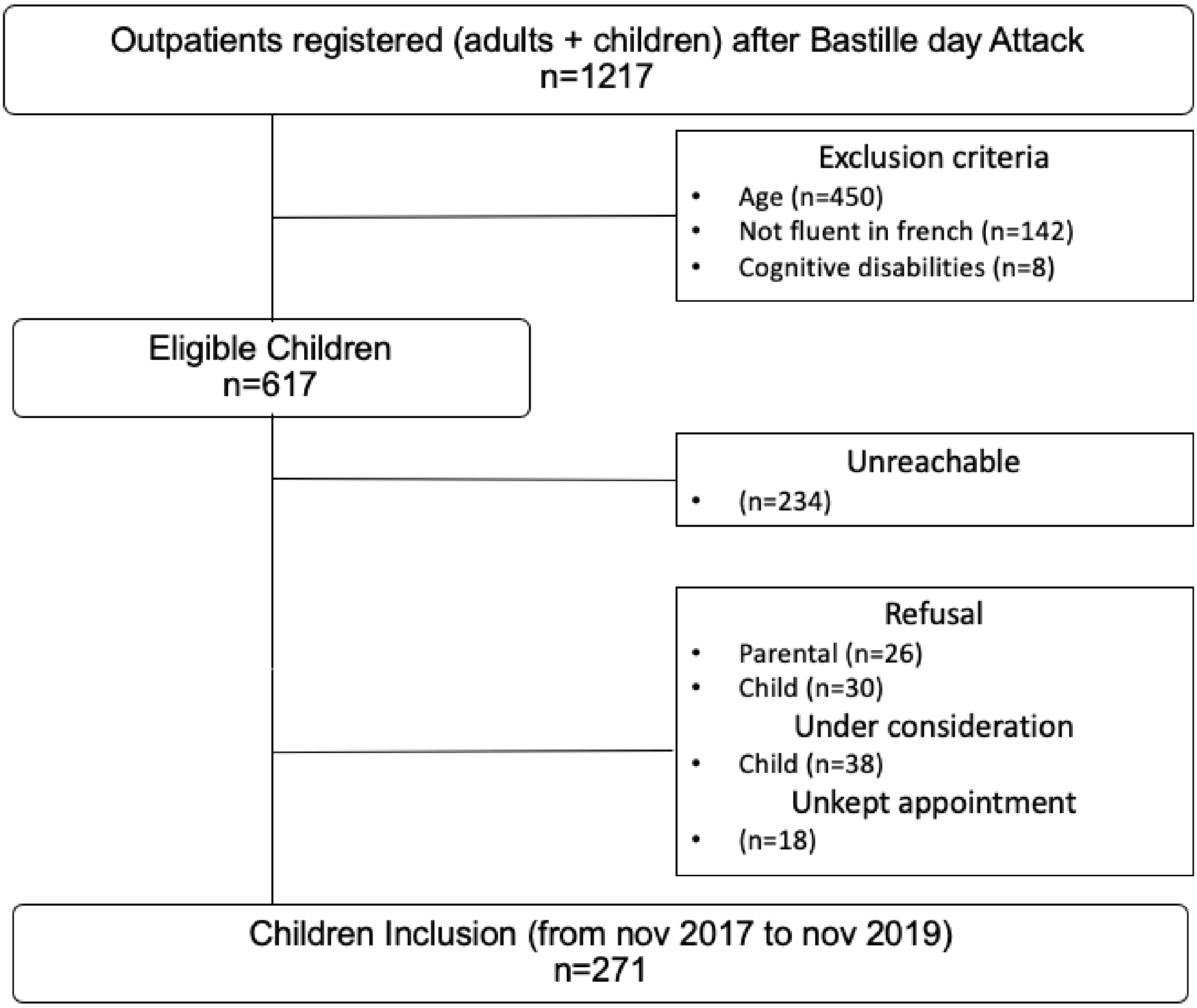

Legend: unreachable patient had change phone number; under consideration patient are still within the legal delay of considering their participation into the study

© 2023 Askenazy F et al. *JAMA Network Open.*
