# Supplementary material for: Analysis of Psychiatric Disorders by Age Among Children Following a Mass Terrorist Attack in Nice, France, on Bastille Day, 2016
Source: JAMA Netw Open. 2023 Feb 3;6(2):e2255472. doi: 10.1001/jamanetworkopen.2022.55472 (PMC9898818; doi:10.1001/jamanetworkopen.2022.55472)
Supplement: Supplement 2. — Data Sharing Statement [file jamanetwopen-e2255472-s002.pdf]

Data Sharing Statement

Askenazy. Analysis of Psychiatric Disorders by Age Among Children Following a Mass Terrorist Attack in Nice, France, on Bastille Day, 2016. *JAMA Netw Open.* Published February 03, 2023. doi:10.1001/jamanetworkopen.2022.55472

Data
Data available: Yes
Data types: Deidentified participant data, Participant data with identifiers, Data (not involving human participants), Data dictionary
How to access data: gindt.m@pediatrie-chulenval-nice.fr
When available: With publication

Supporting Documents
Document types: Informed consent form
How to access documents: gindt.m@pediatrie-chulenval-nice.fr
When available: With publication

Additional Information
Who can access the data: anyone requesting the data
Types of analyses: for any purpose
Mechanisms of data availability: with a signed data access agreement
